# The mitogenomes of *Leptographium aureum*, *Leptographium* sp., and *Grosmannia fruticeta*: expansion by introns

**DOI:** 10.3389/fmicb.2023.1240407

**Published:** 2023-08-10

**Authors:** Jigeesha Mukhopadhyay, Alvan Wai, Georg Hausner

**Affiliations:** Department of Microbiology, University of Manitoba, Winnipeg, MB, Canada

**Keywords:** *Leptographium*, Ophiostomatales, mobile introns, phylogeny, complex introns

## Abstract

**Introduction:**

Many members of the Ophiostomatales are of economic importance as they are bark-beetle associates and causative agents for blue stain on timber and in some instances contribute towards tree mortality. The taxonomy of these fungi has been challenging due to the convergent evolution of many traits associated with insect dispersal and a limited number of morphological characters that happen to be highly pleomorphic. This study examines the mitochondrial genomes for three members of *Leptographium sensu lato* [*Leptographium aureum* (also known as *Grosmannia aurea*), *Grosmannia fruticeta* (also known as *Leptographium fruticetum*), and *Leptographium* sp. WIN(M)1376)].

**Methods:**

Illumina sequencing combined with gene and intron annotations and phylogenetic analysis were performed.

**Results:**

Sequence analysis showed that gene content and gene synteny are conserved but mitochondrial genome sizes were variable: *G. fruticeta* at 63,821 bp, *Leptographium* sp. WIN(M)1376 at 81,823 bp and *L. aureum* at 104,547 bp. The variation in size is due to the number of introns and intron-associated open reading frames. Phylogenetic analysis of currently available mitochondrial genomes for members of the Ophiostomatales supports currently accepted generic arrangements within this order and specifically supports the separation of members with Leptographium-like conidiophores into two genera, with *L. aureum* grouping with *Leptographium* and *G. fruticeta* aligning with *Grosmannia*.

**Discussion:**

Mitochondrial genomes are promising sequences for resolving evolutionary relationships within the Ophiostomatales.

## Introduction

Members of the Ophiostomatales are usually characterized by producing ascocarps with asci that are randomly produced at the base of the ascocarp, and these asci deliquesce with the ascospores being released as a sticky droplet at the tip of the perithecial necks. In addition, many members produce slimy/sticky conidia on long-stalked conidiophores. These are morphological features that facilitate the dispersal of their spores by arthropods (Six and Wingfield, 2011). Historically, the genera *Leptographium* and *Grosmannia* have been assigned to the Ophiostomatales with *Leptographium* accommodating anamorphic (mitotic) members and *Grosmannia* housing those species that have a *Leptographium* anamorph plus a sexual state (reviewed in Jacobs and Wingfield, 2001, 2013, Zipfel et al., 2006). With the introduction of the single name nomenclature as proposed by Hawksworth (2011), *Leptographium*, as the older generic name, should have priority. However, the position of some lineages of fungi with Leptographium-like anamorphs has not been resolved within the Ophiostomatales (Zipfel et al., 2006; de Beer and Wingfield, 2013; Jacobs and Wingfield, 2013; Jankowiak et al., 2018; de Beer et al., 2022). Recent studies showed that there are at least two clades (with the corresponding type specimens: *Leptographium lundbergii* and *Grosmannia penicillata*) that accommodate members of the Ophiostomatales with Leptographium-like conidial states, validating the genera *Leptographium* and *Grosmannia* (de Beer and Wingfield, 2013; de Beer et al., 2022).

*Leptographium aureum* (Rob.-Jeffr. & R.W. Davidson) M.J. Wingf. was isolated from bark beetle-infested, blue-stained pine (Robinson-Jeffrey and Davidson, 1968). In recent surveys, they have been isolated from the aggressive *Dendroctonus ponderosae* (Mountain pine beetle, MPB in western North America; McAllister et al., 2018) and less aggressive *D. murrayanae* (lodgepole pine beetle) beetle in British Columbia Canada (Six et al., 2011). This species was formerly designated as *Europhium aureum*, *Ophiostoma aureum*, and *Grosmannia aureum*, and more recently placed within *Leptographium* (reviewed in Zipfel et al., 2006, and de Beer et al., 2022), demonstrating the complexity and volatility of the taxonomy of this group of fungi. Most members of *Leptographium*/*Grosmannia* are potential agents of blue stain on timber and therefore of economic concerns to the forestry industry (Uzunovic and Byrne, 2013). Some *Leptographium*/*Grosmannia* species are associated with tree diseases (Harrington, 1988; Eckhardt et al., 2004; Jacobs et al., 2004; Hausner et al., 2005) and there is always the potential of moving blue stain fungi into new regions due to the export of infected lumber/timber products and/or the movement/migration of their bark beetle vectors (Jacobs et al., 2004; Hausner et al., 2005; Humble and Allen, 2006). *Grosmannia fruticeta* (Alamouti, J.J. Kim & C. Breuil) M.L. Yin, Z.W. de Beer & M.J. Wingf. has been isolated from *Ips perturbatus* (northern spruce engraver bark beetle) in Northern British Columbia and the Yukon (Massoumi Alamouti et al., 2006, 2007). Frequently exotic fungi are cryptic in the early stages of their invasion and due to morphologically similar appearing native species they are hard to be identified and thus fail to be detected (Santini and Migliorini, 2022). Introduced fungal pathogens can have a severe ecological and economic impact. Therefore, it is important to monitor the movement of native and exotic fungi (Trollip et al., 2021). Biosecurity of forestry resources requires the development of accurate identification strategies. There is considerable interest to generate molecular data that can be used to identify potential plant pathogens or fungi of economic concerns using genomic approaches (Aylward et al., 2017; Trollip et al., 2021, 2022). Kulik et al. (2020, 2021) argued that fungal mitogenomes contain variable regions that could provide a source for molecular markers suitable for fungal identification.

Mitogenomes for the filamentous members of the Ascomycota show great variation in size although encoding a similar set of core genes: *rnl* and *rns* (large and small subunit RNAs involved in protein translation), *cob* and *cox1–3* (coding for components of the respiratory chain complexes), *atp6*, *atp8*, *atp9*, *nad1–6* and *nad4*L (coding for NADH dehydrogenase subunits) and a set of tRNA genes. Sometimes, the ribosomal protein RPS3 (*rps3*; Hausner, 2003; Wai et al., 2019) can be encoded within an *rnl* intron or be found as a free-standing gene. Fungal mitogenome architecture is variable as the results of various recombination events promoted by repeats and by the presence and activities of mobile elements such group I and group II introns and intron-encoded proteins (IEPs) such as homing endonucleases (HEs) and reverse transcriptases (RTs) that facilitate intron mobility and mitochondrial DNA (mtDNA) architecture (Hausner, 2012; Aguileta et al., 2014; Wu and Hao, 2014, 2019; Franco et al., 2017; Repar and Warnecke, 2017; Kulik et al., 2020; Tan et al., 2022). Beyond phylogenetic applications, fungal mitochondrial genomes harbor genetic elements (ribozymes, complex introns (i.e., potentially co-operating ribozymes), and intron-encoded proteins such as endonucleases with novel cutting sites, reverse transcriptases) that have applications in biotechnology as genome editing tools and/or regulatory switches to control gene expression (Takeuchi et al., 2011; Guha et al., 2017; Belfort and Lambowitz, 2019). There is also considerable interest in developing antifungal compounds that target group I introns (Zhang et al., 2000; Malbert et al., 2023).

Currently, only two mitogenomes (mtDNA) have been described for the group *Leptographium/Grosmannia*, these are for *Leptographium lundbergii* and *Grosmannia penicillata* (Zubaer et al., 2021). In this study, we characterize the mitogenomes for the following strains: *L. aureum* (WIN(M)809), *Leptographium* sp. (WIN(M)1376), and *Grosmannia fruticeta* (WIN(M)1600). This work is part of our ongoing effort to study the evolution of mitogenomes for members of the Ophiostomatales (Abboud et al., 2018; Zubaer et al., 2018, 2021; Wai and Hausner, 2021, 2022) with the potential of gaining more insight into the evolution and systematics of these fungi.

## Materials and methods

### Source of culture, culturing methods, and extraction of nucleic acids

Cultures of *Leptographium aureum* CBS 438.69 [ex-Type, (CBS = CBS-KNAW culture collection, Uppsalalaan 8, 3,584 CT, Utrecht, Netherlands); = WIN(M)809; = UAMH 12546 (UAMH = Centre for Global Microfungal Biodiversity, University of Toronto, 223 College St., Toronto ON, Canada M5T 1R4)], *Leptographium* sp. WIN(M)1376 = J.R. 88-194A; = UAMH 12547, and *Grosmannia fruticeta* WIN(M)1600 (= UAMH 12545) were maintained on malt extract agar (MEA; supplemented with yeast extract, 30 g/L malt extract, 1 g/L yeast extract, 20 g/L agar) slants and agar plates (containing approximately 40 mL MEA) and incubated in the dark at 20°C for up to 2 weeks. For the extraction of nucleic acids, three 250 mL Erlenmeyer flasks containing 80 mL of PYG + ME broth (1 g/L peptone, 1 g/L yeast extract, 2 g/L D-glucose, and 3 g/L malt extract) were each inoculated with ten agar blocks (2 mm × 2 mm × 1 mm) and incubated in the dark at 20°C for up to 10 days. Fungal mycelium was harvested from liquid media by vacuum filtration through a Whatman^®^ Grade 1 filter paper in a Büchner funnel. Mycelium was ground up in a pre-chilled mortar with a pestle and acid-washed sand and the DNA for next generation sequencing was recovered and purified as previously described (Wai and Hausner, 2021). DNA samples (30 ng/μL DNA in a final volume of 100 μL) were sent to MicrobesNG (Units 1–2 First Floor, The BioHub, Birmingham Research Park, 97 Vincent Drive, Birmingham, B15 2SQ, United Kingdom) for Illumina sequencing.

### Assembly, analyses, and annotation of next generation sequencing data

Initial analyses of the Illumina reads were performed on the online server GALAXY (Afgan et al., 2018), specifically, the data was uploaded onto Galaxy Europe (usegalaxy.eu). The reads from MicrobesNG were initially assessed using FastQC v0.11.9.^1^ Assemblies were generated by using two programs: SPAdes vs. 3.14.0 (setting the “--careful” option and assembly graph option) and the A5-miseq pipeline (with “-end” option set to “5”; Tritt et al., 2012; Coil et al., 2015). NCBI BLAST + blastn (Camacho et al., 2009) was used to search for sequences of interest (rDNA internal transcribed spacer regions, ITS; and beta tubulin sequences) including contigs/scaffolds corresponding to fungal mtDNAs in all assemblies. In addition to GALAXY, the program Bandage (Wick et al., 2015) was used to examine the assembly graph files generated from SPAdes to aid in the recovery of potential mitogenome contigs. To more efficiently recover mtDNA derived reads and mitochondrial genome assemblies, the program GetOrganelle (Jin et al., 2020) v1.7.5, with the organelle type set to fungus mitogenome (i.e., -F fungus_mt) was applied to the Illumina sequencing reads. In some instances, for *L. aureum*, a set of contigs were recovered, and gaps and/or regions with low coverage were completed/validated by Sanger sequencing PCR products based on primers designed manually (custom designed primers to accommodate for AT-richness of sequences) to bind to the 5′ and 3′ ends of the recovered linear contigs (or regions of low coverage; Supplementary Table 1).

The mtDNA contigs were annotated using the MFannot program (Lang et al., 2023; setting “Genetic Code” to 4)^2^ and RNAweasel^3^; setting to predict tRNAs, and group I (Gr I) and group II (Gr II) introns. Predictions of tRNAs were also performed with tRNAscan-SE 2.0 (Chan and Lowe, 2019). Annotations of protein-coding genes (*atp6*, *atp8*, *atp9*, *cob*, *cox1*–*3*, *nad1*–*6*, *nad4L*, *rps3*) and nonstructural genes (i.e., *rnl* and *rns*, and the tRNAs), were verified by comparative sequence analysis from data obtained from GenBank (Benson et al., 2013). Sequence alignments were generated for all protein coding genes with *Tolypocladium inflatum* serving as a reference genome for naming introns according to the proposed nomenclature by Zhang and Zhang (2019). The rDNA sequences were compared with those of *E. coli* regarding intron annotations (naming) according to Johansen and Haugen (2001). Gene annotations were refined with Artemis (Rutherford et al., 2000) and the mtDNAs were visualized using Circos (Krzywinski et al., 2009). Circos was set up with the appropriate coordinates to highlight exon/intron configurations for conserved protein-coding genes, nonstructural genes, and GC content. The GC plot was generated using a window size of 100 bp and a step size of 20 bp.

The final annotated versions of the mtDNAs characterized in this study have been deposited in GenBank (GenBank accession numbers as follows: for strain WIN(M)809: OQ851464; for strain WIN(M)1600: OQ851465; and for strain WIN(M)1376: OQ851466).

### Phylogenetic analysis of mitochondrial protein coding regions

For the mitochondrial genomes a dataset was generated composed of 13 concatenated amino acid sequences, derived from the following protein-coding genes: *atp6*, *atp8*, *cob*, *cox1*–*3*, *nad1*–*6*, *nad4L*, and data were aligned using MAFFT version 7 (Katoh and Standley, 2013). Some members of the Ophiostomatales do not encode *atp9* within their mitogenomes and so it was not included in the dataset (Zubaer et al., 2021). Additional mitogenomes were obtained from GenBank and the Sequence Read Archive (SRA; Shumway et al., 2010; Leinonen et al., 2011). For more information on the collection, processing, and analyses of the additional mitogenomes, see Zubaer et al. (2021) and Wai and Hausner (2021). In total, 56 mitogenomes were included in the analysis and the tree was visualized and edited using FigTree version 1.4.4.^4^ The root was placed on the branch that splits the Eurotiales and Sordariomycetes and the outgroup consisted of two Eurotiales, *Aspergillus fumigatus* and *Penicillium digitatum*.

The alignment was manually adjusted with AliView version 1.25 (Larsson, 2014) and analyzed with MrBayes 3.2.7a (Huelsenbeck and Ronquist, 2001; Ronquist et al., 2012) for inferring phylogenetic trees. A fixed-rate amino acid substitution model was estimated using the model jumping (i.e., mixed model) function implemented in MrBayes (MB). Rate variation among sites was modeled with a combination of the invariable sites model and gamma model (i.e., rates from a gamma distribution). Other parameters were left at their default values (i.e., a uniform distribution between 0.0 and 1.0 and an exponential distribution with a mean value of 1.0, respectively). The analysis was performed with 2,000,000 generations with a sampling frequency of 1,000. The cpREV model was estimated with the highest probability. The first set of 25% of sampled trees was discarded (burn-in) and the remaining trees were used to construct the 50% majority rule consensus tree. The aligned data set was also analyzed with programs contained within MEGA XI (Tamura et al., 2021): Maximum Likelihood (ML). For ML, the LG model (plus I and F) was applied, and 1,000 bootstrap replicas were analyzed to assess branch support values.

### Phylogenetic analysis of nuclear markers: its and beta tubulin

Sequences were collected from NCBI (GenBank) and from whole genome assemblies for the internal transcribed spacer (ITS) region (GenBank: OR146620 to OR146622) and partial beta-tubuline (βT) gene sequences (GenBank: OR146617-OR146619) to generate a potential species tree for members of *Leptographium*. The data were aligned with MAFFT, and phylogenetic trees were generated with MEGA XI (ML, model T92 + G) and MrBayes (MB, setting mixed model converging with GTR + I + G, 1,000,000 generations, sample frequency at 100, and burnin value at 30%).

## Results

### The mitogenomes: size variation and gene content

**Figure 1:**
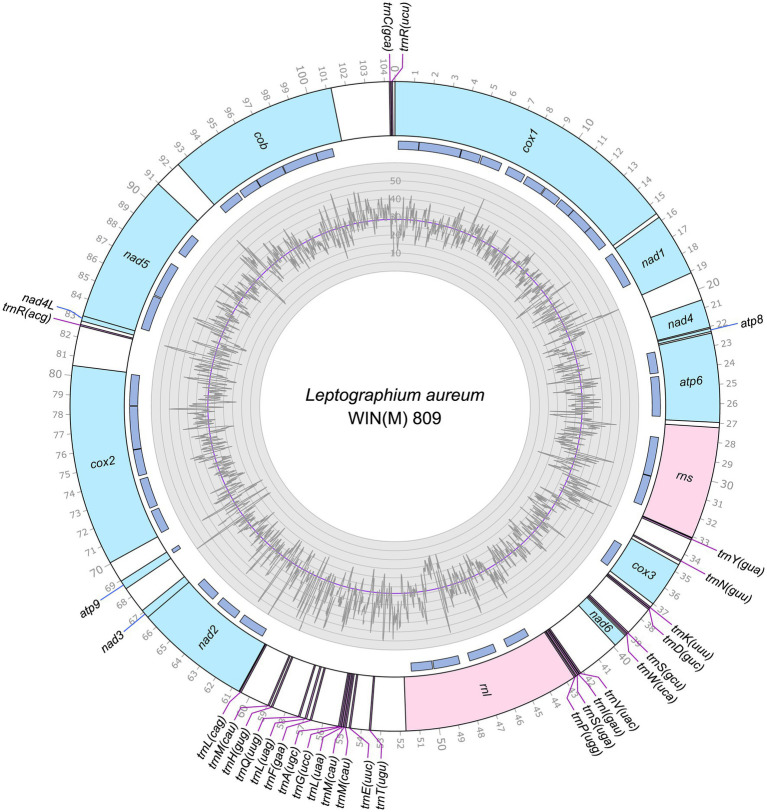
Circular representation of the mitochondrial genomes of *Leptographium aureum* WIN(M)809 (GenBank accession number: OQ851464). Genes, introns, and GC plot are shown on the outer, middle, and inner tracks, respectively. The purple line of the GC plot corresponds to the average GC content of the mitochondrial genomes. The tick marks on the outer track label every 1,000th nucleotides, starting from the beginning of the *cox1* gene. All labeled genes are encoded on the same strand.

**Figure 2:**
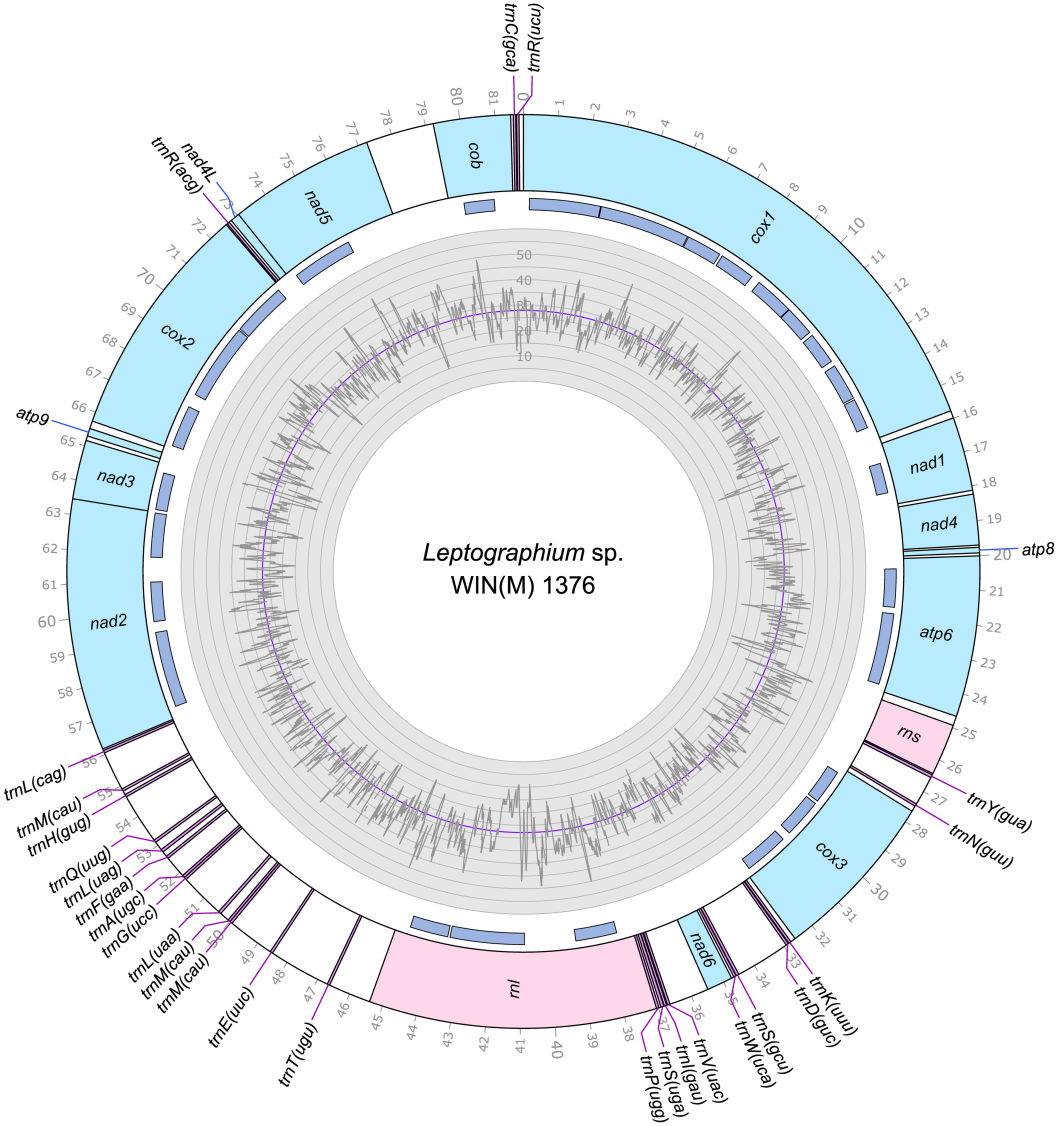
Circular representation of the mitochondrial genomes of *Leptographium* sp. WIN(M)1376 (GenBank accession number: OQ851466). Genes, introns, and GC plot are shown on the outer, middle, and inner tracks, respectively. The purple line of the GC plot corresponds to the average GC content of the mitochondrial genomes. The tick marks on the outer track label every 1,000th nucleotides, starting from the beginning of the *cox1* gene. All labeled genes are encoded on the same strand.

**Figure 3:**
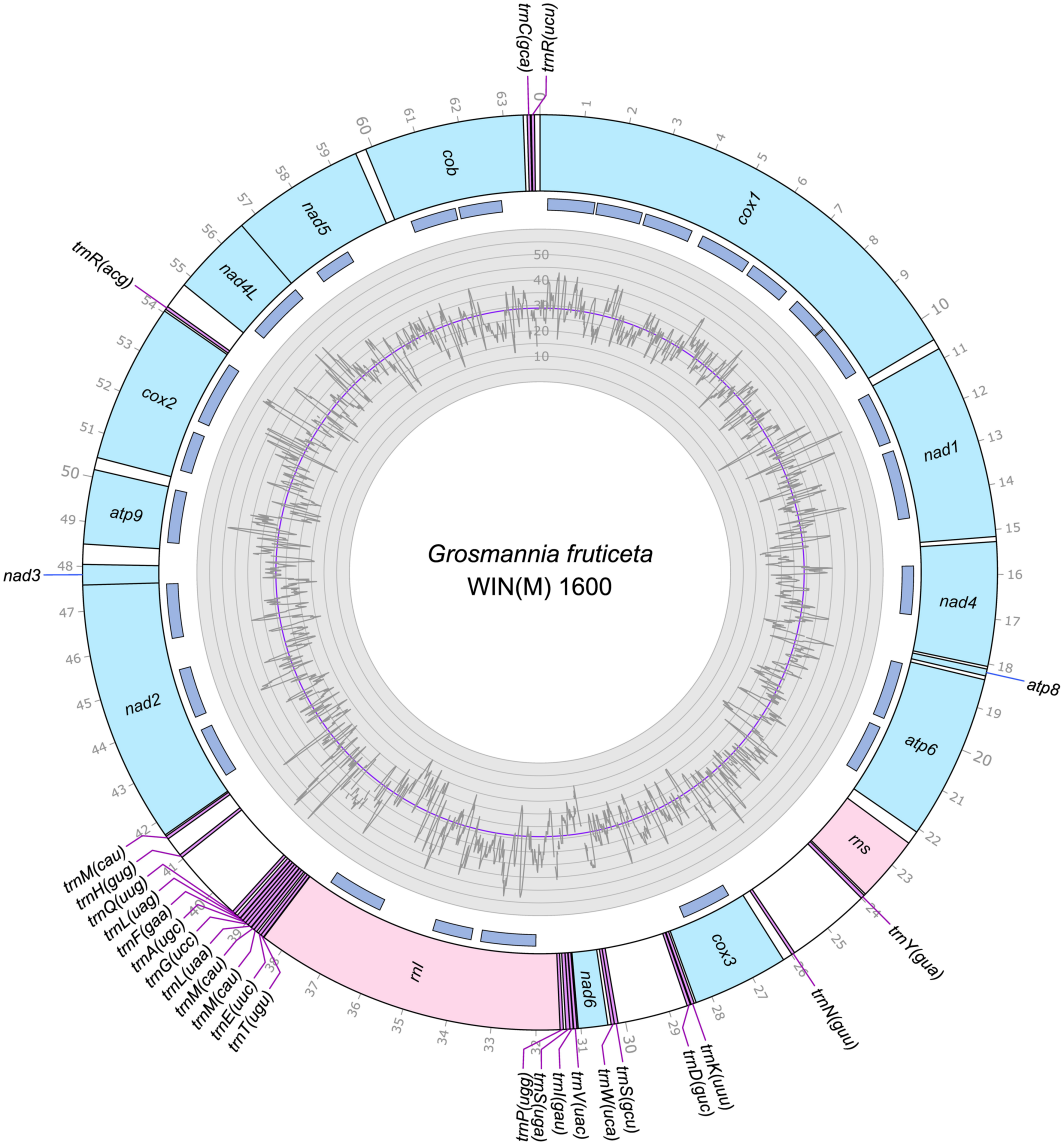
Circular representation of the mitochondrial genomes of *Grosmannia fruticeta* WIN(M)1600 (GenBank accession number: OQ851465). Genes, introns, and GC plot are shown on the outer, middle, and inner tracks, respectively. The purple line of the GC plot corresponds to the average GC content of the mitochondrial genomes. The tick marks on the outer track label every 1,000th nucleotides, starting from the beginning of the *cox1* gene. All labeled genes are encoded on the same strand.

**Figure 4:**
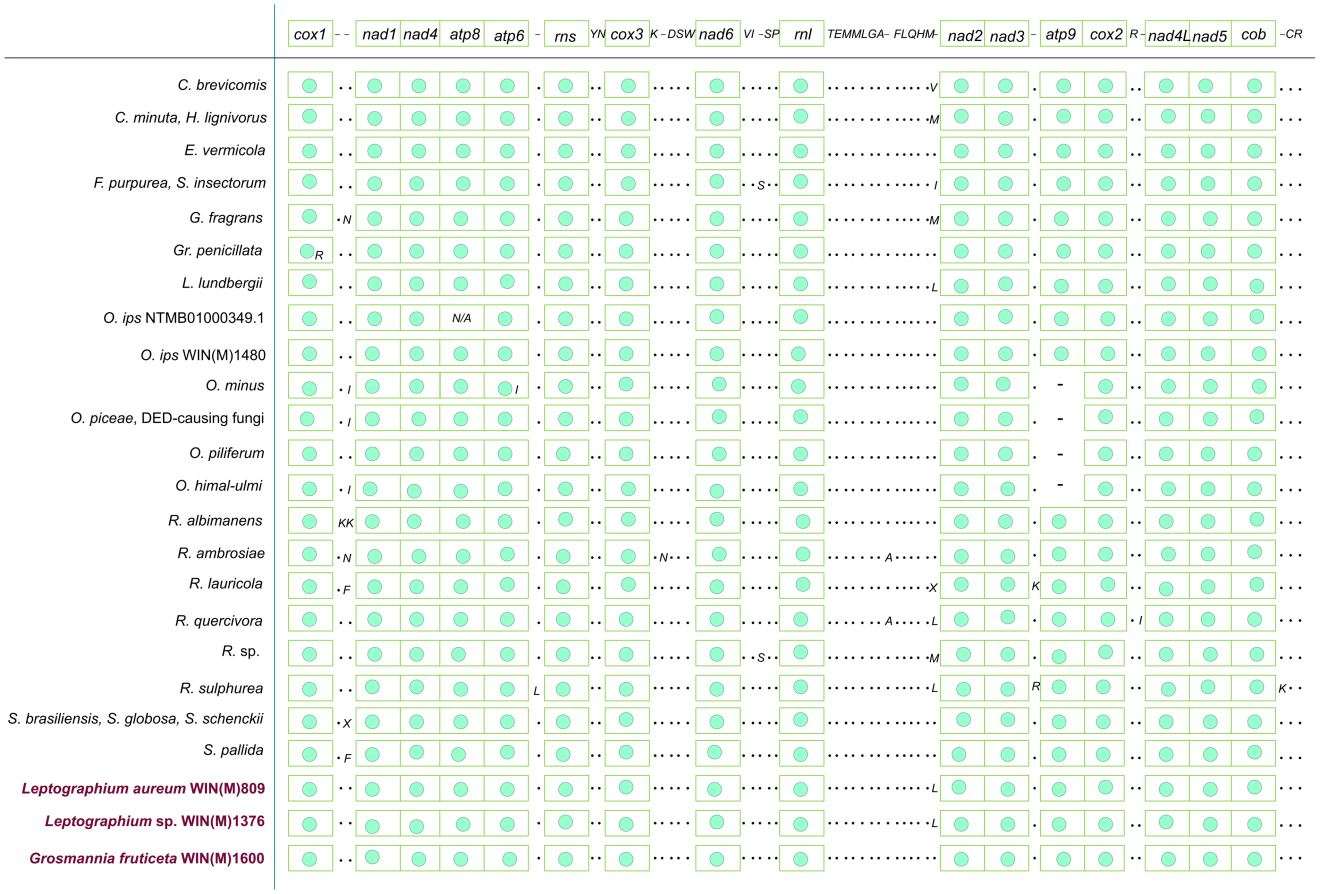
Gene synteny for 26 members of the Ophiostomatales. Amino acids are represented with the single-letter code. C., *Ceratocystiopsis*; E., *Esteya*; F., *Fragosphaeria*; G., *Graphilbum*; Gr., *Grosmannia*; L., *Leptographium*; O., *Ophiostoma*; R., *Raffaelea*; S., *Sporothrix*; “N/A,” not applicable; “–“, absence of gene; “·,” presence of tRNA genes and “green circle,” presence of ribosomal and protein-coding genes (as in first line). The presence of *atp9* is observed for the three species reported in this study. Among the reported species of *Ophiostoma*, only *Ophiostoma ips* is known to encode *atp9* in the mitogenome. Variation in terms of presence/absence is observed mostly for tRNA genes among the Ophiostomatales.

The mitogenomes of *L. aureum* WIN(M)809, *Leptographium* sp. WIN(M)1376, and *Grosmannia fruticeta* WIN(M)1600, are represented as circular molecules and are illustrated in Figures 1–3, respectively. These vary in size with *L. aureum* at 104,547 bp, *L.* sp. WIN(M)1376 at 81,823 bp, and *G. fruticeta* at 63,821 bp. Based on the program GetOrganelle vs. 1.7.5 the coverage for the three mitogenomes were as follows: 2,491 fold for *L. aureum*, 1,555 fold for WIN(M)1376, and 1,409 fold for *G. fruticeta*. All three genomes encode the ribosomal RNA genes *rns* and *rnl*, the mitochondrial core set of protein-coding genes (*atp6*, *atp8*, *atp9*, *cob*, *cox1*–*3*, *nad1*–*6*, *nad4L*, and *rps3*). Like other members of the Ophiostomatales (and members of the Sordariomycetes) the ribosomal protein RPS3 is encoded within a Gr I intron embedded within the *rnl* gene (reviewed in Wai et al., 2019). The presence of *atp9* was noted for all three genomes and these also encode a set of tRNAs that cover all 20 standard amino acids. The mitogenomes for *L. aureum* and *L.* sp. WIN(M)1376 encode 26 tRNA genes and the *G. fruticeta* genome encodes 25 tRNAs genes. The gene order and orientation for all three genomes is identical and the same as previously reported for members of the Ophiostomatales (Wai and Hausner, 2021; Zubaer et al., 2021): *cox1*, *nad1*, *nad4*, *atp8*, *atp6*, *rns*, *cox3*, *nad6*, *rnl*, *nad2*, *nad3*, *atp9*, *cox2*, *nad4L*, *nad5*, *cob* (Figure 4). For *L. aureum* and *L.* sp. WIN(M)1376 the *nad4L* and *nad5* genes are separated by one nucleotide and the *nad2* and *nad3* genes are “fused” although they are separated by a stop (TAA) codon. In *G. fruticeta* the *nad4L* and *nad5* genes overlap by one nucleotide and the *nad2* and *nad3* genes are fused as described for *L. aureum* and *L.* sp. WIN(M)1376.

The tRNA genes are arranged into a few interspersed clusters with the majority of tRNAs arranged upstream and downstream of the *rnl* gene and a grouping of four tRNA genes between the *cox3* and *nad6* genes. *L. aureum*, *L.* sp. WIN(M)1376, and *G. fruticeta* have the same tRNA gene configuration except for the larger tRNA cluster downstream of the *rnl* gene, here the *G. fruticeta* has 12 instead of 13 tRNA genes and it has a *trnL* as its last member for this cluster compared to *trnM* as seen in the other two species. This is the same as was observed previously for *G. penicillata* which has the *trnL* as its terminal member for this tRNA gene cluster whereas *L. lundbergii* has the *trnM* gene at this location (Figure 4; Zubaer et al., 2021). Most mitochondrial tRNA genes are single copies but there are exceptions. All three species have three copies of *trnM* (CAU), *L. aureum* and *L.* sp. WIN(M)1376 have alternate versions for *trnL* (CAG, UAG and UAA), *trnR* (ACG and UCU) and *trnS* (GCU, UGA) and *G. fruticeta* has the same alternate versions for *trnL*, *trnR* and *trnS* except it lacks *trnL* (CAG).

### Intron content of the studied mitogenomes

The mitochondrial genomes of *L. aureum*, *L.* sp. WIN(M)1376 and *G. fruticeta* contain 37, 27 and 25 introns, respectively. In *L. aureum*, 36 are Gr I introns, encoding double-motif LAGLIDADG (LAG(2)) and GIY-YIG (GIY) type open reading frame (ORF) and one Gr II intron that encodes a reverse transcriptase-like (RT) protein (see Figure 4). In *L.* sp. WIN(M)1376 and *G. fruticeta*, all the introns can be assigned to Gr I, encoding LAG(2) and GIY ORFs. In some fungal mitogenomes, the *rnl* group IA (*rnl-*2,450; nomenclature based on Johansen and Haugen, 2001) intron encodes the RPS3 protein (Bullerwell et al., 2000). The *rnl*-2,450 intron in *L. aureum* encodes a LAG(2) (ORF434) located downstream of the rps3 ORF, whereas in *L*. sp. WIN(M)1376 the *rnl*-2,450 encoded ORF is a fusion of the rps3 coding sequence with a LAG(2) coding sequence. In *G. fruticeta* WIN(M)1600 the *rnl*-2,450 intron only codes for RPS3.

**Figure 5:**
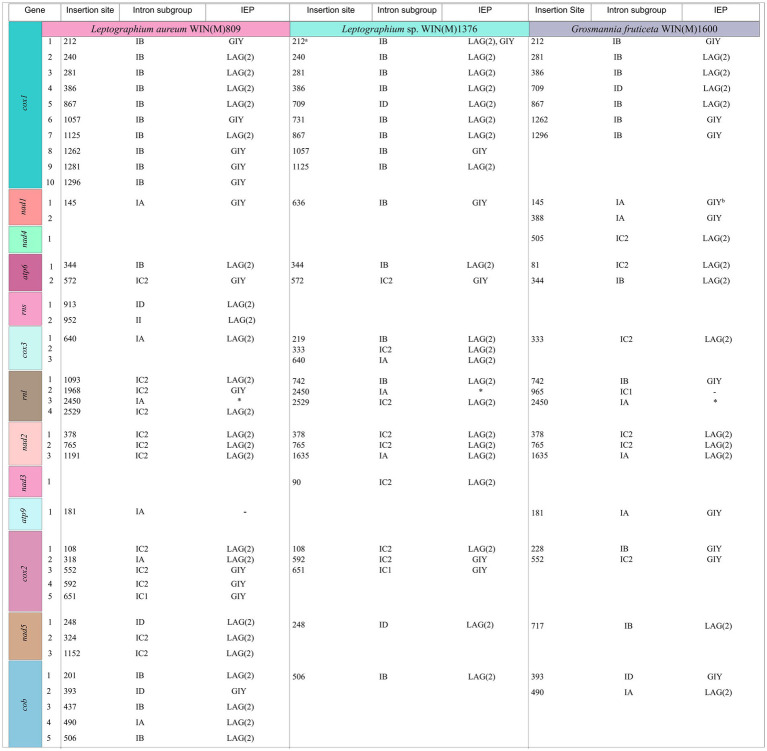
Summary of introns and intron-encoded proteins observed within the mitochondrial genomes of *Leptographium aureum* WIN(M)809, *Leptographium* sp. WIN(M)1376 and *Grosmannia fruticeta* WIN(M)1600. Intron insertion sites for protein coding genes were based on *Tolypocladium inflatum* (NC_036382.1; Zhang and Zhang, 2019). No introns have been identified for *nad6* and *atp8* genes. Intron subgrouping applies only to group I introns; group II introns are annotated as “II”; Unidentifiable = No identifiable group I or II intron based on MFannot/RNAweasel results and manual inspection. Intron-encoded protein (IEP) associated with intron insertion site based on BLASTx results; LAG(2), double-motif LAGLIDADG homing endonuclease; RT, reverse transcriptase; GIY, GIY-YIG homing endonuclease; “–“, no conserved motif detected based on BLASTx results. ^a^In WIN(M) 1,376, *cox1*-212 is a complex intron with one complete IB and a partial IB intron module. Both positioned at 212 with reference to *T. inflatum*. The second intron module is speculated to have invaded the resident intron module. ^b^Appears to encode a degenerate GIY. ^*^Appears to encode a “rps3/HE-like fusion protein” (Bullerwell et al., 2000).

**Figure 6:**
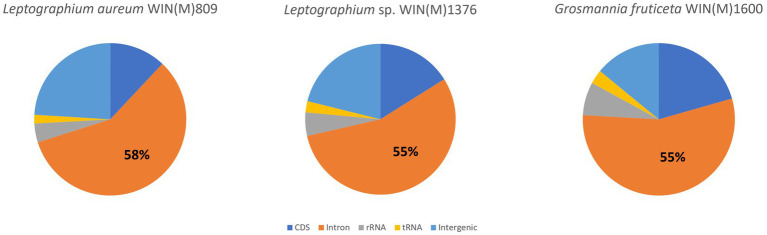
Composition of the mitochondrial genomes of *Leptographium aureum* WIN(M)809 (1a), *Leptographium* sp. WIN(M)1376 (1b) and *Grosmannia fruticeta* WIN(M)1600 (1c) showing the proportion of introns (including intron-encoded ORFs).

Figure 5 summarizes the location of all introns, their classification, and the intron-encoded proteins (IEPs). The *atp9* group IA intron in *L. aureum* and *rnl* Gr IC1 intron in *G. fruticeta* show no evidence for an ORF (introns *atp9*-181 and *rnl*-965, respectively). The *nad1* Gr IA intron (*nad1-*145) appears to encode a degenerate GIY ORF. In *L. aureum*, *Leptographium* sp. WIN(M)1376 and *G. fruticeta* the *cox1* gene has the most intron insertions (10, 9 and 7, respectively) followed by the *cox2* (5) and *cob* (5) genes in WIN(M)809 which is the most intron-rich genome in this (Figure 5). Introns and intergenic sequences accounted for most of the genome size (> 80%), with 58% intron content in *L. aureum* and 55% intron content in *L.* sp. WIN(M)1376 and *G. fruticeta* (Figures 6A–C).

**Figure 7:**
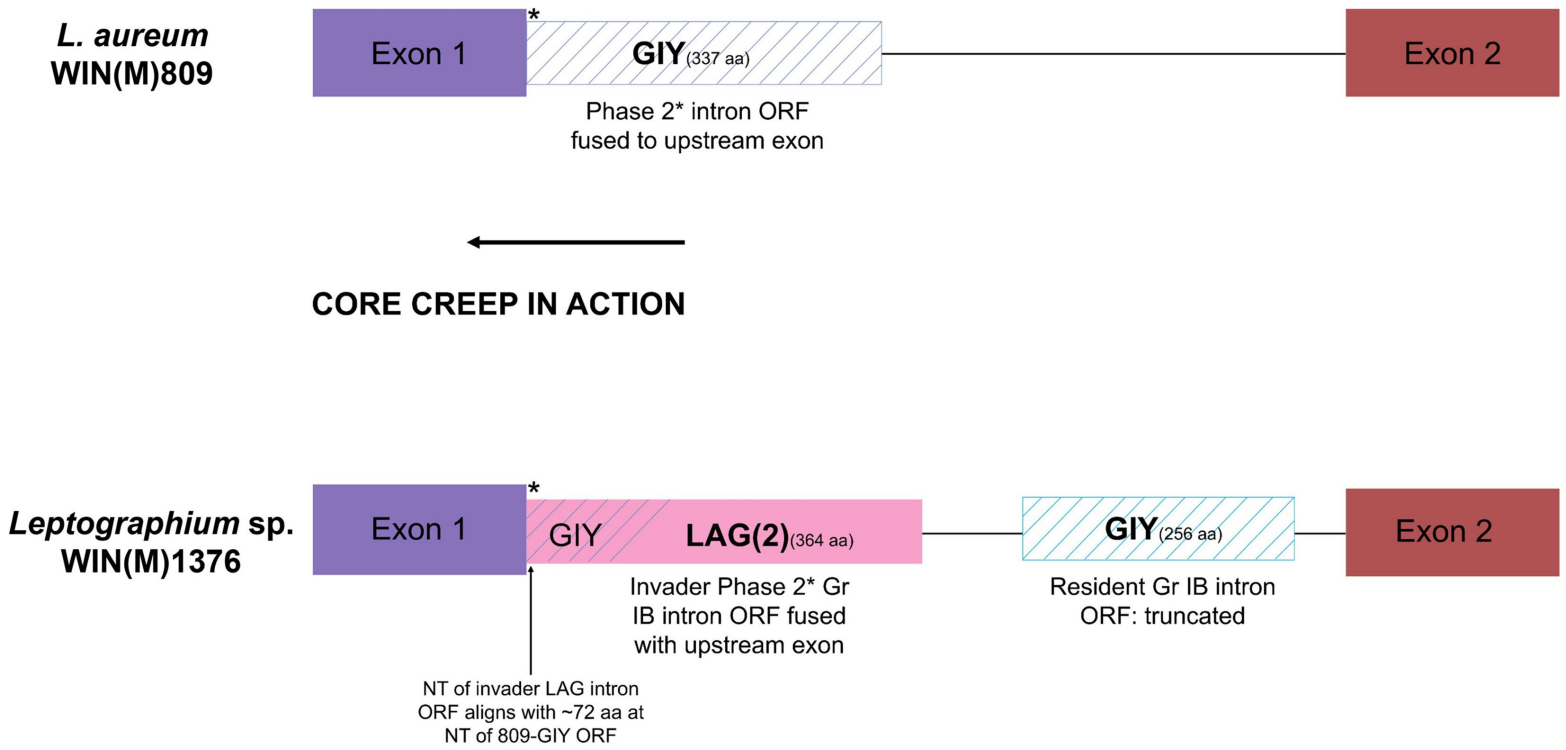
Schematic representation of the *cox1*-212 intron of *L. aureum* WIN(M)809 and *Leptographium* sp. WIN(M)1376. The *cox1*-212 intron in *L. aureum* belongs to the subgroup Group I and is a phase 2 intron (interrupts the second position of the codon) as the intron ORF (GIY) is fused to the upstream exon (see Figure 5). Similarly, the first intron in the *cox1* gene in WIN(M)1376 is also a phase 2 intron (subgroup IB) where the LAG(2) ORF is fused with the upstream exon. It is speculated that this is the result of ‘core creep’ where the intron ORF over time, has incorporated upstream intronic sequences to fuse in-frame to the upstream exon (Edgell et al., 2011; Mukhopadhyay and Hausner, 2021). This fusion would allow the IEP to be more efficiently expressed, as it gains regulatory sequences of the host gene that optimize translation. The N-terminus of the LAG(2) ORF in *L.* sp. WIN(M)1376 coincides with the N-terminus of the GIY intron in *L. aureum*, indicating that the remaining part of the resident GIY ORF has been displaced downstream due to the invasion of the LAG(2) intron in *L.* sp. WIN(M)1376.There also is a partial IB intron component in *cox1-*212 intron of *L.* sp. WIN(M)1376.

A complex intron can be recognized in the *cox1* gene at position 212 (based on Gr I intron naming nomenclature) in *L.* sp. WIN(M)1376. A related version (same insertion site) of this intron was also recorded for *L. aureum* but here the intron is a Gr IB intron encoding a GIY ORF that is fused (in frame) to the upstream *cox1* exon. In *L.* sp. WIN(M)1376 the intron is composed of a Gr IB module encoding a GIY YIG ORF and a partial Gr IB intron module that appears to be located within the P1 region of the host Gr IB intron (see Figure 7). Based on sequence comparison it appears that the original version, as represented by the *cox1*-212 intron in *L. aureum*, was invaded by a LAG(2)-type homing endonuclease gene (HEG) that moved along with it a partial Gr I B intron sequence. The LAG(2) HEG inserted into the N-terminal region of the resident GIY YIG ORF, fused in frame with the GIY component and thereby is in frame with the upstream *cox1* exon. The *cox1*-212 intron in *G. fruticeta* is composed of a Gr IB intron encoding a degenerated (fragmented) version of a GIY ORF due to the presence of premature stop codons.

### Phylogenetic analysis of the mitochondrial genomes

**Figure 8:**
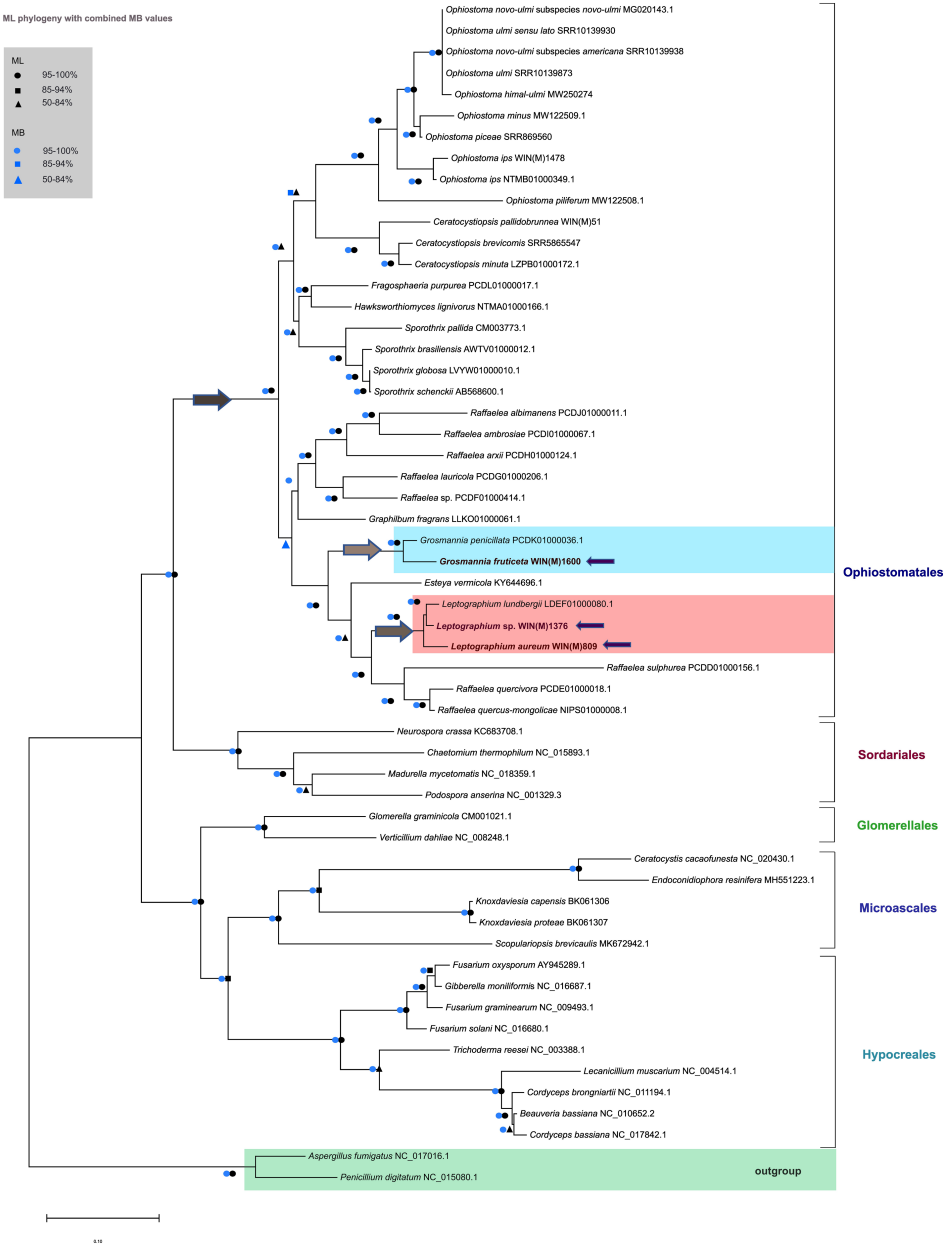
Phylogenetic relationships of 56 fungal species belonging to the Ascomycota are presented, based on concatenated amino acid sequences, composed of *atp6*, *atp8*, *cob*, *cox1*, *cox2*, *cox3*, *nad1*, *nad2*, *nad3*, *nad4*, *nad4L*, *nad5*, and *nad6*. *Aspergillus fumigatus* and *Penicillium digitatum* are selected as the outgroups. Maximum Likelihood (LG + I + F) as implemented in MEGA XI was used to generate the phylogenetic tree using the bootstrap option (1,000 pseudoreplicates) to estimate node support values. A second tree was constructed based on Bayesian inference and the posterior probability (PP) support values from the 50% majority Bayesian (MB) consensus tree are represented in combination with the Maximum Likelihood node support values (bootstrap support) on this tree. Key nodes of interest are indicated by arrows; dark grey arrow shows the node branching into the Ophiostomatales; light grey arrows indicate the nodes of branching into *Grosmannia* and *Leptographium* groups. The three fungal species in this study are also indicated by purple arrows. Black circles, squares and triangles represent bootstrap support (95–100%), (85–94%), and (50–84%) respectively for the ML analysis while blue circles, squares and triangles correspond to the PP support (95–100%), (85–94%), and (50–84%) respectively for the MB analysis, at the branches. Branch lengths are proportional to the number of substitutions per site (see scale bar).

The mitogenome based phylogeny is based on the dataset comprised of 56 sequences that include 34 sequences representing 12 of the currently accepted 14 genera of the Ophiostomatales (de Beer et al., 2022). All members of the Ophiostomatales can be derived from one branch with high levels of confidence (100%) based on ML and MB analysis (Figure 8). The inferred phylogeny yielded a topology that supported the monophyly of the following groupings: Microascales, Hypocreales, Glomerellales, Sordariales, and the Ophiostomatales. Among the Ophiostomatales, we were able to sample from 12 genera: *Ophiostoma*, *Sporothrix*, *Ceratocystiopsis*, *Fragosphaeria*, *Hawksworthiomyces*, *Raffaelea*, *Harringtonia* (previously referred to as the *Raffaelea lauricola* complex; de Beer and Wingfield, 2013, de Beer et al., 2022), *Graphilbum*, *Grosmannia*, *Esteya*, *Leptographium*, and *Dryadomyces* (now including the *Raffaelea sulphurea* complex; de Beer and Wingfield, 2013; de Beer et al., 2022).

The mitogenome-based tree agrees with phylogenies published based on nuclear markers (reviewed in de Beer et al., 2022). The *L. aureum* and *L.* sp. WIN(M)1376 sequences grouped with *L. lundbergii* and *G. fruticeta* grouped with *G. penicillata*. With regards to *L. aureum*, *Leptographium* sp. WIN(M)1376, and *G. fruticeta* the mitogenomes confirm phylogenetic placements as obtained from the analysis of nuclear rDNA ITS regions and partial beta tubulin sequences (Supplementary Figures 1A,B; image 1 and 2 respectively). The mitogenome data does confirm the separation of species with Leptographium-like conidial states into the two genera *Leptographium* and *Grosmannia*.

## Discussion

### Mitochondrial genome organization

Mitochondria contain their own genomes and they have been reported to be derived from alpha-proteobacteria through endosymbiosis (Muñoz-Gómez et al., 2017). Compared with the more conservative genome sizes in animals, fungal mitogenomes show a wide variation in size and differ from their animal counterparts in terms of lower substitution rates, presence of introns and associated mobile elements, higher noncoding DNA, and recombination-associated repair mechanisms (Hausner, 2012; Freel et al., 2015; Deng et al., 2018; Lang, 2018; Sandor et al., 2018; Fonseca et al., 2021). Fungal mitochondrial genomes can be presented as a single circular molecule or be linear concatemers composed of mtDNA units in tandem arrangements (Valach et al., 2011; Lang, 2018). Although fungi share a common set of mitochondrial core genes, there is high variability in terms of gene order among the fungi, both between and within the major phyla (i.e., basidiomycetes, ascomycetes, and early diverging fungi; Aguileta et al., 2014).

Among the Ophiostomatales, mitogenomes can vary greatly in size ranging from 23.7 kb to about 150 kb (Zhang et al., 2019; Wai and Hausner, 2021; Zubaer et al., 2021). Size variation among the Ophiostomatales mitogenomes is in part due to the absence/presence of potential mobile introns, intron-encoded open reading frames, intergenic spacers, duplications, presence of non-conserved (or sometimes referred to as unknown) open reading frames (non-conserved or ncORFs and unidentified or uORFs; Wai and Hausner, 2021; Zubaer et al., 2021). Organellar introns can be either Gr I or Gr II introns, differentiated by their splicing mechanisms and their secondary and tertiary folds at the RNA level. The introns can encode open reading frames (ORFs) that express proteins that may catalyze their mobility to cognate alleles and/or enhance splicing efficiency by acting as maturases (reviewed by Prince et al., 2022).

Although mitochondrial gene synteny is highly conserved among the Ophiostomatales, the main variation arises with respect to the tRNA genes and the presence or absence of *atp9*. For the three fungal species studied, shared tRNA synteny was observed for *L. aureum* WIN(M)809 and *L.* sp. WIN(M)1376 with *L. lundbergii* whereas *G. fruticeta* WIN(M)1600 shared tRNA synteny with *Esteya vermicola* (Figure 4) and for all these mitogenomes, the presence of *atp9* was noted. The absence of the *atp9* gene has been observed in other fungal taxa and presumable nuclear mitochondrial-derived versions of *atp9* have been identified Déquard-Chablat et al. (2011), Franco et al. (2017), Zubaer et al. (2018), Wai and Hausner (2021), and Zaccaron et al. (2021) suggesting that within the fungi a copy of the mitochondrial *atp9* gene was transferred to the nuclear genome. A study by Déquard-Chablat et al. (2011) demonstrated that many fungi have one or more copies of nuclear-encoded version(s) of *atp9* that encode the ATP9 peptide component for the mitochondrial ATP synthase. Scanning the nuclear genome contigs for *L. aureum*, *G. fruticeta*, and *L*. sp. WIN(M) identified nuclear-encoded *atp9* genes (GenBank accession numbers: OR271575-OR271577) therefore it can be assumed that members of the Ophiostomatales have nuclear-encoded versions of *atp9* and in some instances the mitochondrial version of *atp9* has been lost (see Zubaer et al., 2019; Wai and Hausner, 2021).

In fungi, most genes related to mitochondrial function are found in the nuclear genome (Bolender et al., 2008). It has been speculated that the escape of the mtDNA involves transfer to the cytosol and, consequently, to the nucleus where these genetic fragments are integrated with the aid by mobile elements that use the non-homologous (NHEJ) machinery (Berg and Kurland, 2000; Tsuji et al., 2012; Korovesi et al., 2018). The selective advantage of mtDNA transfer to the nucleus has been attributed to escaping the build-up of deleterious mutations, protection of DNA from mitochondrial mutagens (oxygen radicals) and thus the fixing of beneficial mutations (Allen and Raven, 1996; Blanchard and Lynch, 2000; Saccone et al., 2000). However, a core set of protein coding genes, rRNA genes and tRNA genes are retained in the mitochondrial genome for efficient local control of energy metabolism (Lang et al., 2012; Allen, 2015).

Other common gene arrangements among fungi include the fusion of *nad2* and *nad3* genes and the overlap between the ORFs of the *nad4L* and *nad5* genes. *L. aureum* and *L.* sp. WIN(M)1376 are closely related to *L. lundbergii* and *G. fruticeta* is closely related to *G. penicillata*, and these related members of the Ophiostomatales show the fusion of *nad2-nad3* genes. As for the *nad4L-nad5* ORF arrangements, there is one nucleotide (T) separation in *L. lundbergii,* and one nucleotide overlap in *G. penicillata* (as observed in *G. fruticeta*). In the latter, the overlap of the stop and initiation codons between these genes in these pairs is the cause for their contiguity.

The tRNA gene clusters noted in the studied fungi (Figure 4) have also been observed in other members of the Ophiostomatales such as the tRNA genes reported upstream and downstream of the *rnl* gene (Wai and Hausner, 2021). In some metazoan mitogenomes, it has been proposed that tRNAs are positioned in a pattern that promotes the resolution of polycistronic mRNAs, referred to as the punctuation model (Salinas-Giegé et al., 2015). In the fungi examined in this work, not all genes are separated by tRNAs so the punctuation model may not apply to the Ophiostomatales. The tRNA clusters near the rDNA genes (*rns* and *rnl*) ma**y** ensure all RNA molecules needed for translation are expressed at appropriate amounts.

### Genome expansion due to intron proliferation

The three members of *Leptographium sensu lato* in this study were reported to have intron-rich genomes with introns and intergenic regions covering >80% of the mitogenome size, reinforcing their role in generating mitochondrial genome diversity. Related members such as *G. penicillata* at 150.9 kb (64 introns) that groups with *G. fruticeta* groups and *L. lundbergii* at 101.8 kb (36 introns) that groups with *L. aureum* and *L.* sp. WIN(M)1376 also boast of intron-rich genomes correlated to their large genome sizes. This is consistent across different fungal phyla with variable genome sizes, sometimes associated with large-scale gene rearrangements, and complex intron dynamics (Zubaer et al., 2018, 2021; Li et al., 2020, 2021, 2023), indicating that phylogenetic positions do not necessarily correlate with genome size or intron content. One example of significant mitogenomic collinearity and consistent mitochondrial gene arrangement with vastly different mitogenome sizes has been reported for two members of the Pleosporales, with *Exserohilium rostratum* genome at 64,620 bp housing 17 introns (and 17 intronic ORFs) while the closely related *Exserohilium turcicum* at 264,948 bp contained 70 introns (and 126 intronic ORFs). This great difference in intron number suggests that gain/loss events of introns frequently occur in the mitogenome evolution in these fungi (Ma et al., 2022).

The variation in intron numbers among fungal mitochondrial genomes is puzzling with examples of streamlining (intron-loss) and genome expansions driven by intron-gain among the various members of the Ophiostomatales (Abboud et al., 2018; Zubaer et al., 2021). Introns and intergenic DNA in mitogenomes were originally considered a genetic liability, as they are targets for deleterious and potentially lethal mutations (Lynch et al., 2006). The mutational burden hypothesis (MBH) was postulated to explain the origin of organellar genome size (Lynch et al., 2006) which stated that introns and intergenic DNAs tend to accumulate when natural selection is less efficient at purging hazardous non-coding DNA (Xiao et al., 2017).

Elevated sequence evolution near mobile introns and increased density of single nucleotide polymorphisms (SNP) in exon regions approaching intron boundaries have been noted in yeast mitogenomes (Repar and Warnecke, 2017), which correlate with homing endonuclease recognition sites. The rapid turnover of mobile introns can significantly impact genome size, but there are only a limited number of available intron insertion sites due to the requirement of conserved sequences for homing endonuclease. Thus, the expansion and contraction of mitogenomes (due to the gain and loss of introns) may cause only subtle change per event, but they take place persistently within the space limit (reviewed in Hao, 2022).

The presence and influence of non-coding elements in mitogenomes have been explored across several fungal groups (Fonseca et al., 2020; Araújo et al., 2021; Kulik et al., 2021). Models have been proposed that argue that mobile introns and their mobility-promoting IEPs are examples of “neutral evolution” (Goddard and Burt, 1999; reviewed in Hao, 2022). The model is supported by examples of introns and associated ORFs that invade a site followed by degeneration of the ORF, intron, and eventual loss of the element, which generates an “empty site” that can be reinvaded. Lack of selection permits a cycle of invasion, degeneration, loss, and reinvasion. Homing endonuclease genes (which can be free-standing or embedded within introns) are classified as selfish or parasitic elements that can transpose by breaking the DNA at specific sites, which consequently leads to gene conversion (Barzel et al., 2011; Stoddard, 2014). Mobile introns can move horizontally and invade new genomes, facilitated by IEPs (HEs for Gr I introns and reverse transcriptases for Gr II introns; reviewed by Hausner, 2012, Mukhopadhyay and Hausner, 2021). Intron and HEG origins and their coevolution have been speculated (Loizos et al., 1994; Megarioti and Kouvelis, 2020) and it has been postulated that introns serve as a “safe haven” for these HEGs to get established. Edgell (2009) states that both HEGs and introns are selfish, independent elements, but they often benefit from being in close genomic proximity (e.g., HEGs are established by introns, and introns can use the encoded endonuclease to spread more easily). The association of HEGs with introns ensures their mutual persistence in the genome, evading the negative selective pressure to eliminate selfish elements from the genome and thus outpacing degeneration with mobility to unoccupied (cognate or new) sites by outcrossing and other means (Stoddard, 2014). Transient hyphal fusion might allow the exchange of cytoplasm between different fungal strains that could lead to fusion of mitochondria to facilitate mtDNA recombination and homing or ectopic transposition of introns and HEGs. Intron loss can be mitigated by intron components (ribozyme or ORF) gaining essential functions such as acting as trans-acting maturases or the intron encoding essential proteins (such as RPS3).

Although initially viewed as autocatalytic, organellar introns typically require cofactors to achieve splicing-competent configurations, these can be intron- or nuclear-encoded (Lang et al., 2007; Prince et al., 2022). The requirement of additional components can turn these introns into regulatory elements whose removal becomes a rate-limiting step in the expression of their host genes. Rudan et al. (2018) suggested that mitochondrial intron splicing is an essential component for normal mitochondrial function in *Saccharomyces cerevisiae*. If this is true for other fungi, the splicing of mitochondrial introns and their reliance on cis- and trans-acting factors encoded by the mitochondrial and nuclear genomes may be a fortuitous mechanism that evolved to allow for finetuning mitochondrial function to specific environments and life histories. For members of the Ophiostomatales, mtDNA intron landscapes have been generated and biases with regards to intron insertion sites and genes that are more likely to be intron-rich, have been noted, but there are no conserved introns found in all members of this group of fungi (except for *rnl*-2450 that encodes for RPS3; Zubaer et al., 2021; Wai and Hausner, 2022). This might imply that fine-tuning mitochondrial gene regulation is not based on specific introns, instead based on an assemblage (or intron complement) composed of various introns (at various sites) that are “functionally” redundant. Finally, the reliance on nuclear factors for organellar intron splicing impacts mitonuclear compatibilities (or incompatibilities) and potentially imposes reproductive barriers, thereby promoting speciation events (reviewed in Dujon, 2020).

Complex introns are composed of multiple intron modules, which may be the result of introns invading other introns. Twintron-like arrangements have been identified in fungal mitochondrial genes, which splice out in consequent inter-dependent steps or as zombies (Zumkeller et al., 2020) where the composite intron splices as one unit. High intron load may promote more recombination events, but it may also place constraints on those exon sequences that are required for proper intron splicing (P1 and P10 interactions). Horizontal transfer among closely related (or distant) species of introns in addition to co-conversion of flanking markers promotes variability among members of the population. Thus, complex introns could serve as platforms for alternative splicing pathways that can be explored in terms of their potential in differential expression patterns for their associated ORFs. In this study, a complex intron was predicted in the *cox1* gene of *L.* sp. WIN(M)1376 where a double-motif LAGLIDADG HEG invaded a resident Gr I intron ORF and brought along a partial Gr IB intron segment, fused in-frame with the GIY ORF, and the upstream *cox1* intron. This fusion is an example of “core creep” (Edgell et al., 2011; Mukhopadhyay and Hausner, 2021) whereby the intronic ORF extended (“creeped”) towards the upstream exon and has fused in frame with the exon (Edgell et al., 2011). This pseudo-“exonization” event (intron sequences becoming coding sequences, but eventually spliced out) requires that the intron RNA folding capacity is not altered due to changes at the nucleotide level that result in the intron ORF being fused to the upstream exon. This might facilitate the intron-encoded ORFs that are present in protein-coding genes to be more efficiently translated. In the same instance “core creep” is achieved by alternate splicing, here transcripts are generated that fuse the intron-encoded reading frame with the upstream exon segment (Turk et al., 2013; Sellem et al., 2016; Guha et al., 2018; Zubaer et al., 2018, 2021; Deng et al., 2020).

### Phylogenetic analysis of mitogenomes representing various genera of the Ophiostomatales

Comparative mitogenomics has been applied to elucidate the population structure and genomic organization of fungal species, and study factors related to fungal pathogenicity, production of substances and enzymes of commercial interest, and other aspects of fungal evolution and biology including their coevolution with their associated vectors (Stajich, 2017; Jankowiak et al., 2018). The taxonomy of the Ophiostomatales has undergone considerable revisions in recent years; currently, the order Ophiostomatales includes two families, Kathistaceae and the Ophiostomataceae and monophyly is often not certain for the groupings *Leptographium sensu lato* (includes *Grosmannia* species) and *Ophiostoma sensu lato* (de Beer and Wingfield, 2013; de Beer et al., 2016). In this study, phylogenetic analysis relying on both DNA-based markers (ITS, beta tubulin) and mitogenome-derived amino acid sequences helped in confirming the separation of *Leptographium* and *Grosmannia* species, with *L. aureum* and *L.* sp. strain WIN(M)1376 grouping with *L. lundbergii* and *G. fruticeta* grouping with *G. penicillata.* In addition, the mitogenome-derived data supports the division of *Raffaelea sensu lato* (de Beer and Wingfield, 2013) into three genera: *Raffaelea*, *Harringtonia*, and *Dryadomyces* (de Beer et al., 2022).

## Conclusions and applications

This study showed that the structural variation and variabilities in size and composition in fungal mitogenomes could mainly be explained by the presence of accessory elements (introns and their encoded products). This study also identified an example of a complex intron and a phenomenon linked to it, referred to as “core creep” where intron encoded ORFs have fused to the upstream exons to enhance the expression of intron encoded proteins. The use of concatenated mitogenome derived amino acid sequence-based phylogenies has the potential in resolving taxonomic groupings within the Ophiostomatales. As more mitogenome sequences and multi-loci sequence data for reference specimens become available for members of the Ophiostomatales, these can serve as a resource for DNA-based markers that can be utilized for large-scale phylogenetic genomic-based diagnostics and species delimitation. This could eventually lead to the development of a platform that can be used to recognize and explore the taxonomic diversity within the Ophiostomatales and assist in developing high-throughput strategies in biosecurity services to detect pathogens and invasive fungi (Trollip et al., 2021, 2022).

## Data availability statement

The datasets presented in this study can be found in online repositories. The names of the repository/repositories and accession number(s) can be found at: https://www.ncbi.nlm.nih.gov/genbank/, OQ851464 https://www.ncbi.nlm.nih.gov/genbank/, OQ851465 https://www.ncbi.nlm.nih.gov/genbank/, OQ851466 https://www.ncbi.nlm.nih.gov/genbank/, OR146620 https://www.ncbi.nlm.nih.gov/genbank/, OR146621 https://www.ncbi.nlm.nih.gov/genbank/, OR146622 https://www.ncbi.nlm.nih.gov/genbank/, OR146617 https://www.ncbi.nlm.nih.gov/genbank/, OR146618 https://www.ncbi.nlm.nih.gov/genbank/, OR146619 https://www.ncbi.nlm.nih.gov/genbank/, OR271575 https://www.ncbi.nlm.nih.gov/genbank/, OR271576 https://www.ncbi.nlm.nih.gov/genbank/, OR271577.

## Author contributions

JM and AW have been working under the supervision of GH and all obtained data and contributed towards the analysis. JM, AW, and GH contributed towards the design of the project, and worked on the manuscript. JM and AW took the lead with regards to assembling the datasets and the final analysis of the data. GH assembled the final version of the manuscript. All authors contributed to the article and approved the submitted version.

## Funding

This work was supported by Natural Sciences and Engineering Research Council of Canada (NSERC) Discovery (Grant: RGPIN-2020-05332).

## Conflict of interest

The authors declare that the research was conducted in the absence of any commercial or financial relationships that could be construed as a potential conflict of interest.

## Publisher’s note

All claims expressed in this article are solely those of the authors and do not necessarily represent those of their affiliated organizations, or those of the publisher, the editors and the reviewers. Any product that may be evaluated in this article, or claim that may be made by its manufacturer, is not guaranteed or endorsed by the publisher.

## Footnotes

^1^ https://www.bioinformatics.babraham.ac.uk/projects/fastqc/

^2^ https://megasun.bch.umontreal.ca/cgi-bin/dev_mfa/mfannotInterface.pl

^3^ https://megasun.bch.umontreal.ca/cgi-bin/RNAweasel/RNAweaselInterface.pl

^4^ http://tree.bio.ed.ac.uk/software/figtree/
