# Supplementary material for: The mitogenomes of Leptographium aureum, Leptographium sp., and Grosmannia fruticeta: expansion by introns
Source: Front Microbiol. 2023 Aug 10;14:1240407. doi: 10.3389/fmicb.2023.1240407 (PMC10448965; doi:10.3389/fmicb.2023.1240407)
Supplement: Supplementary file 1 [file Table_1.DOCX]

Table S1.

Custom primers designed for *Leptographium aureum* WIN(M)809. F= “forward primers”; R= “reverse primers”. The range of positions of primers corresponds to GenBank accession number: OQ851464 for strain WIN(M)809

| Primer name | Sequence | Notes on regions covered |
| --- | --- | --- |
| 809_F1_a | GAGTTAGTGGATATACTAATG | 68674-69148:  between the end of *nad3* and beginning of *atp9* |
| 809_F2_a | GATAGCAGACAAGCATTAG |  |
| 809_R1_a | CCATGTGTTATATGGTG |  |
| 809_R2_a | CTGTGTTGATCAGACTATGTC |  |
| 809_F1_b | GAAGACATAGTCTGATCAACACAG | 69208-70030:  between the end of *atp9* and beginning of *cox2* |
| 809_F2_b | GATGTTACCTATATAGG |  |
| 809_R1_b | CCTCGGAGTTAGAATATTCC |  |
| 809_R2_b | GATGCCGCCGCAAGCCTGC |  |
| 809_F1_c | GCAGGCTTGCGGCGGCATC | 70090-71090:  between the end of *atp9* and beginning of *cox2* |
| 809_F2_c | GAGGTTGAAGTAGTAGATATTG |  |
| 809_R1_c | CATCGAAGAACCGTCTACTCG |  |
| 809_R2_c | CAAGCTTCACCGTCTGAGAAC |  |
| 809_F1_d | AGTACAGGTGTTGTTAC | 91349-92852:  between the end of *nad5* and beginning of *cob* |
| 809_F2_d | GCTTATGTTATTAGGTGG |  |
| 809_R1_d | GAACTTACACTAGTAATG |  |
| 809_R2_d | ACCGTAGGTAATATAATAAG |  |
