# Supplementary figures and images for: The mitogenomes of Leptographium aureum, Leptographium sp., and Grosmannia fruticeta: expansion by introns
Source: Front Microbiol. 2023 Aug 10;14:1240407. doi: 10.3389/fmicb.2023.1240407 (PMC10448965; doi:10.3389/fmicb.2023.1240407)

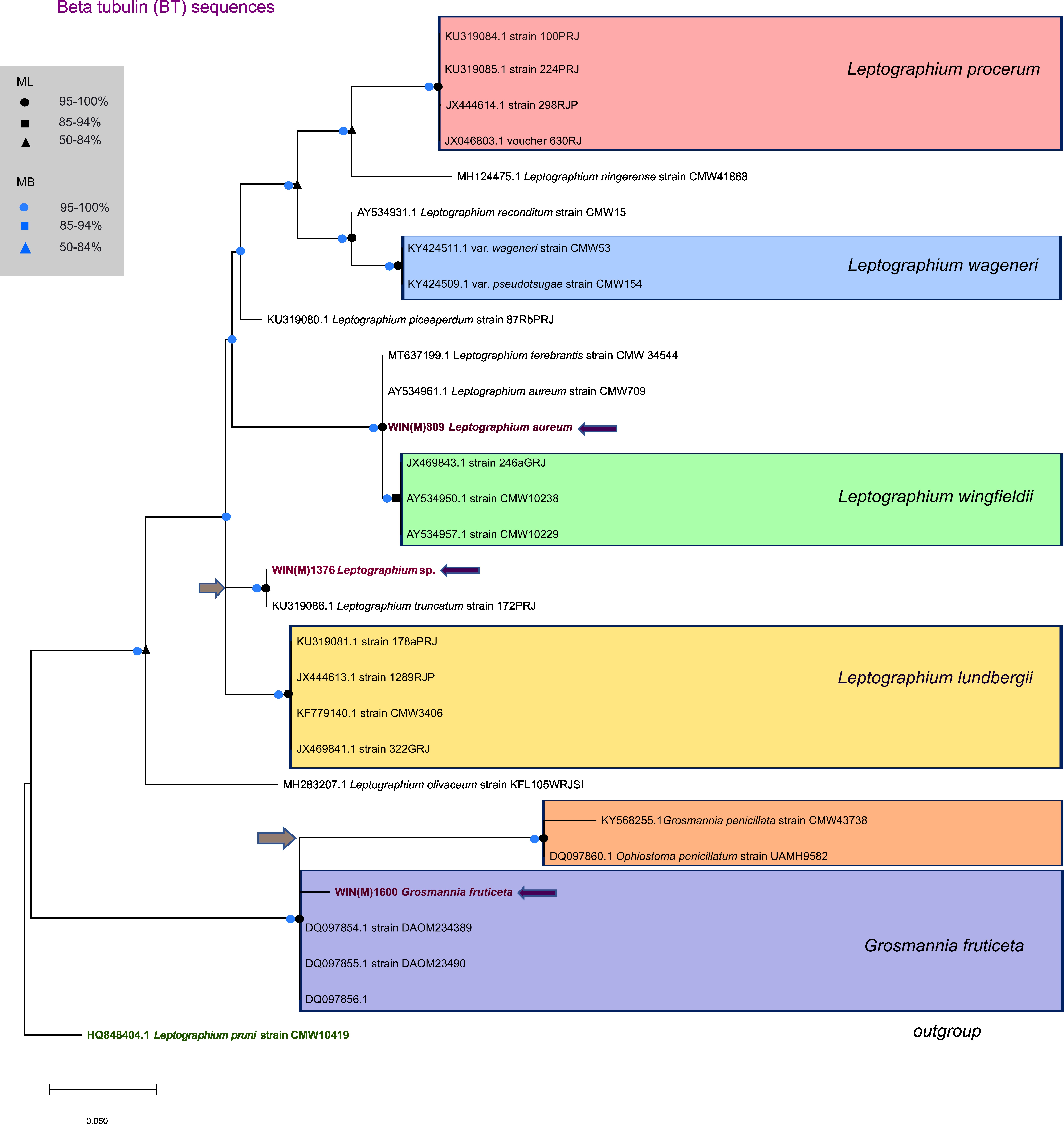

Supplement: Supplementary file 3 [file Image_2.JPEG]
